# Norepinephrine exerts an inotropic effect at the early phase of human septic shock

**DOI:** 10.1186/2197-425X-3-S1-A798

**Published:** 2015-10-01

**Authors:** O Hamzaoui, M Jozwiak, T Geffriaud, B Sztrymf, D Prat, F Jacobs, X Monnet, P Trouiller, C Richard, JL Teboul

**Affiliations:** Paris Sud University, Service de Réanimation Polyvalente, Hôpital Antoine Béclère, Clamart, France; Paris Sud University, Service de Réanimation Médicale, Hôpital de Bicêtre, Le Kremlin-Bicêtre, France

## Introduction

Norepinephrine (NE) is recognized as a potent vasopressor used in septic shock to reverse hypotension. Whether NE still exerts a positive effect on cardiac contractility through beta-adrenergic stimulation is unclear since the sensitivity of beta-adrenergic receptors can be abnormally reduced in septic conditions. Our study was designed to address this issue at the early phase of septic shock.

## Methods

We prospectively included patients suffering from septic shock, with a mean arterial pressure (MAP) < 65 mmHg despite initial hemodynamic resuscitation. Echocardiographic measurements were performed before (T0) and after either initiating NE or increasing its dosage in order to achieve a target MAP >65 mmHg (T1). The following variables were collected: left ventricular ejection fraction (LVEF), velocity-time integral (VTI) of the aortic flow, cardiac output (CO), tissue Doppler imaging of tricuspid annular motion (Sa), peak early transmitral flow velocity (E), tissue Doppler imaging of mean systolic (Sm) and mean early diastolic velocity (Ea) of the lateral mitral annulus and the ratio E/Ea.

## Results

we included 28 patients (mean age of 71 ± 12 years), their mean SAPS 2 was 59 ± 22, the main sites of infection were the lung in 13 patients, the abdomen in 6 patients and the urinary tract in 5 patients. Eighteen patients were mechanically ventilated. The mean interval time between the start of resuscitation and T0 was of 141 ± 72 min. At T0, patients have already received a mean volume of fluids of 1500 ± 700 mL. At T0, 15 patients did not yet receive NE while in the other 13 patients, the mean dose of NE was 0.55 ± 0.62 µg/kg/min. At T1, the mean dose of NE was 0.69 ± 0.62µg/Kg/min for the whole population. From T0 to T1, MAP increased significantly from 56 ± 7 to 80 ± 7 mmHg, diastolic arterial pressures increased from from 44 ± 6 to 59 ± 12 mmHg and lactate decreased from 3.2 ± 2.2 to 2.3 ± 1.8 mmol/L (p < 0.05). From T0 to T1, LVEF increased from 48 ± 15 to 55 ± 14% (p < 0.05), Sm increased from 10.7 ± 5 to 12 ± 5 cm/s (p < 0.05), VTI increased from 18 ± 6 to 20 ± 7 cm (p < 0.05), CO increased from 5.6 ± 1.9 to 6.1 ± 2.5 l/min (p < 0.05), E increased from 82 ± 28 to 94 ± 30 cm/s (p < 0.05), E/Ea increased from 9.3 ± 6 to 10.5 ± 6 (p = 0.05) and Sa increased from 13 ± 6 to 15 ± 7 cm/s (p < 0.05). Even in the 10 patients with a LVEF ≤ 40% at T0, LVEF and VTI significantly increased (from 33 ± 6 to 41 ± 9% and from 16 ± 7 cm to 18 ± 9 cm, respectively).

## Conclusions

In spite of the increase in arterial blood pressure and thus in left ventricular afterload, NE administration at the early phase of septic shock increased the indices of left ventricular systolic function, even when the left ventricular systolic function is altered. This suggests that NE actually exerted a significant inotropic effect at the early phase of septic shock in addition to the effect on preload.

